# The Late Dr. Schorstein and Medical Education

**Published:** 1907-03-02

**Authors:** Samuel Montagu, Lewis McIver, C. H. Dorking, Stephen MacKenzie, William Osler, Percy Kidd

**Affiliations:** London Hospital Medical College, Whitechapel, E.; London Hospital Medical College, Whitechapel, E.; London Hospital Medical College, Whitechapel, E.; London Hospital Medical College, Whitechapel, E.; London Hospital Medical College, Whitechapel, E.; London Hospital Medical College, Whitechapel, E.


### THE LATE DR. SCHORSTEIN AND MEDICAL EDUCATION.

Sir,—We shall be grateful if you will spare us space to advocate simultaneously the claims of medical education at the London Hospital Medical College and a memorial to one of the most prominent physicians at that college.

The colleagues and friends of the late Dr. Schorstein are anxious to perpetuate his memory by the endowment of an advanced course of lectures in clinical medicine, which it has long been recognised is one of the greatest needs of the medical school. It is not intended to create by this a course to form part of the ordinary school curriculum, but rather something on the lines of the endowed lectureships at the Royal College of Physicians, such as the Gouldstonian.

It is proposed that a course or courses of three or four lectures be given by a physician to be elected by the governing body of the school. The number of lectures and the remuneration will be determined by the amount of the money raised; but we wish to make clear (1) that the election to the lectureship will be regarded as a professional distinction; (2) that due notice will be given, so as to afford the lecturer ample time to prepare a discourse worthy of the subject and of the man in whose memory it is given.

Subscriptions, large or small, may be sent to the Hon. Secretaries of the Fund: Dr. Cholmeley, 11 Portland Place, W.; Dr. Cecil Wall, 6 Cavendish Place, W., or to the Manager, Barclay's Bank, 27 Cavendish Square, W. They will be acknowledged on a numbered receipt form; but no list of either donors or amounts will be published, as this, it is felt, would have been in accordance with Dr. Schorstein's wish.

Dr. Cecil Wall will be pleased to supply further details of the scheme on application. We remain, yours truly,

Samuel Montagu, Lewis McIver, C. H. Dorking, Stephen Mackenzie, William Osler, Percy Kidd.

London Hospital Medical College, Whitechapel, E.

